# Supplementary material for: Are carbon emissions trading and green financial instruments synergistic? -Comprehensive quantitative research based on content analysis
Source: PLoS One. 2024 Mar 7;19(3):e0298601. doi: 10.1371/journal.pone.0298601 (PMC10919601; doi:10.1371/journal.pone.0298601)
Supplement: S1 Data — (ZIP) [file pone.0298601.s001.zip › Data/地级市Z_F工作报告.zip!/╣π╬≈/╣≤╕█╩╨/└┤╫╘╣1⁄2╓┌║┼í╛┬φ┐╦╩2╛▌═°í┐_2010.docx]

各位代表：

现在，我代表市人民政府，向大会作政府工作报告，请予审议，并请各位政协委员和其他列席人员提出意见。

2009年工作回顾

2009年是进入新世纪以来我市经济发展遇到困难最多的一年，也是我们经受考验、攻坚克难、逆势而上的一年。一年来，市人民政府在自治区党委、政府和市委的领导下，在市人大及其常委会监督帮助和市政协的大力支持下，团结带领全市各族人民，积极应对国际金融危机的冲击，努力克服持续秋旱的影响，抓好事关改革发展大局的各项关键性措施落实，较好地完成了市三届人大五次会议提出的各项目标任务，经济社会保持了又好又快的发展态势。全年实现地区生产总值438.6亿元，增长15.2％；财政收入34.03亿元，增长17.1%；全社会固定资产投资290.18亿元，增长31.8％；社会消费品零售总额181.1亿元，增长19％；进出口总额1.43亿美元，同比下降12.6%；城镇居民人均可支配收入13915元，增长9.9%；农民人均纯收入4504元，增长11.2％；化学需氧量和二氧化硫排放总量分别削减4.57%、16.88%；规模以上万元工业增加值能耗下降0.75%。

一年来，我们主要做了以下工作：

（一）积极应对各种挑战，力促经济保持平稳较快增长。面对全球金融危机带来的种种困难和不利影响，我们认真贯彻落实中央、自治区一系列决策部署，迎难而上求发展，奋力拼搏保增长。通过举行报告会、座谈会，认清形势，坚定广大干部群众攻坚克难的信心和决心；集中做好国家投资项目的策划、呈报和争取工作，一批谋划多年的重点项目实现开工；优化金融生态环境，深化政银企合作，全年新增贷款64.9亿元；深入基层和企业调研，与各级干部和企业界人士分析困难、研究对策，并及时出台帮扶工业、房地产业、商贸流通业等一系列政策措施，加大生产要素协调力度，千方百计帮助企业解决生产经营中的困难和问题。经过积极努力，全市经济在困难较多的形势下仍然实现快速、均衡、科学发展，基本实现了市人大三届五次会议审议通过的预期目标，主要指标超额完成了市委提出的工作目标任务，总体成效好于预期。

（二）认真落实强农惠农政策，“三农”工作取得新成效。以促进农民增收为核心，围绕“力争三年实现全市农民人均纯收入赶超全国平均水平”的目标，认真挖掘农业发展潜力，加快发展特色农业，切实加强“三农”工作，农业和农村经济保持稳定发展。农林牧渔业总产值实现163.4亿元，增长5.1%。粮食总产量达143.6万吨，增长3.7%，实现4年连续增产。蔬菜、木薯、马铃薯、食用菌、中药材等优势农业规模进一步扩大，新增特色农业基地5万亩。新建标准化规模养殖小区82个，新发展规模养殖户363户，畜禽饲养总量稳定增长。农业产业化加快发展，新增农业产业化重点龙头企业8家、农民专业合作社475家。培训农民38.2万人次，农村劳动力转移就业新增5万人。投入4.98亿元加强水利基础设施建设，对9座水库实施除险加固，硬化灌区渠道104公里，恢复有效灌溉面积2.3万亩，改善有效灌溉面积10.6万亩；建成人饮工程187个，解决农村43.67万人的饮水不安全问题。新建沼气池8960座。重视改善水库移民生产生活条件，投入5000万元建成水库移民进村（屯）道路工程99个、移民新村35个。认真落实农机购置补贴政策，农业机械化水平明显提高。集体林权制度改革扎实推进，超额完成造林绿化任务。启动实施城乡风貌改造试点，新农村建设取得了新成绩。

（三）着力扶持企业度难关，工业经济质量和效益大幅回升。采取财政贴息、减免税费等一系列切实可行措施，加强对贵糖、金源化工等重点企业的扶持，重视帮助中小企业化解不利因素，工业经济实现逆势攀升。规模以上工业增加值实现112.96亿元，增长25.6％；实现利润10.5亿元，增长257.9%。新增规模以上工业企业45家。建材、冶金、电力、化工、机械等支柱产业保持平稳较快发展，工业结构进一步优化。工业技改投资完成69.6亿元，增长34.8%。园区经济进一步壮大，园区工业增加值39.5亿元，增长29.5%。着力抓好节能减排，实施了7个工程减排项目，关停了一批不符合国家产业政策、污染严重的企业。

（四）不断拓宽服务业领域，第三产业发展水平明显提高。第三产业增加值实现159.3亿元，增长15%。水运货物周转量和公路货物周转量分别增长21.6%、26.7%；港口货物吞吐量完成3390万吨，增长8.9%，其中集装箱吞吐量完成6万标准箱，增长19.3%。“万村千乡”市场工程和“新网工程”扎实推进，新建和改造农家店370家，农家店覆盖73%的行政村。旅游产业逆势增长，全年接待游客总人数和旅游总收入分别增长17%、43%。桂平市获得“国家地质公园”称号。房地产业健康发展，完成投资33.6亿元，实现税收2亿元，分别增长22.7%、30.4%。保险、信息、中介、社区服务等新兴服务业都得到较快发展。

（五） 深入实施项目带动战略，发展后劲进一步增强。认真开展“项目建设年”活动，着力抓好投资上项目工作，保持投资对经济增长的强劲拉动作用。全市新开工规模以上项目116个，完成投资70.44亿元，华润（平南）三期、史丹利复合肥项目一期、清隆机械扩建等57个项目竣工投产。南广高速铁路建设进度加快，梧州至贵港、桂平（石龙）至来宾高速公路开工建设。大藤峡水利枢纽、平南白沙核电等重大项目前期工作扎实推进。中央扩大内需投资的264个项目全部按计划开工。交通固定资产投资完成21.7亿元，覃塘至凤凰二级公路全线竣工通车，完成通乡通村水泥路项目58个370公里，其中通村水泥路项目17个114公里。郁江北堤建设加快推进，郁江南堤项目实现开工。投入8亿元加快电网基础设施建设，瑞雪、鹤岭等一批重大电网项目竣工投入使用。对外开放进一步扩大，承接产业转移成效显著。新引进合作项目426个，实际利用市外资金143亿元，增长47%；实际利用外资1.35亿美元，增长2%；外贸出口1.1亿美元，增长16.9%。海关入库税款2.3亿元，增长43.2%。对外交流有了新的发展，我市与韩国首尔市九老区签订友好城市意向。成功举办西江流域经济发展论坛，与沿江各市达成《贵港共识》。

（六）加快城镇化建设，城乡面貌进一步改善。突出新区建设和旧城改造并进，中心城区建成区面积扩大到48.9平方公里，全市城镇化率提高到35.6%。城市总体规划修编和土地利用总体规划修编通过专家评审，港口总体规划获自治区同意并呈报国家交通运输部候批。投入64亿元加强城市基础设施建设，中心城区重点城建项目建设加快，马草江生态公园对市民开放，南山公园改造基本完成，市图书馆、市博物馆、荷城初中开工建设，迎宾大道北延段、荷城路西延段竣工通车，新开工建设布山路西延段、新区三路、南山路等9条城市道路。在中心城区新建公厕20座、垃圾转运站5座，完成小街小巷改造200条。新增城市公共绿地77.21公顷，城区绿化覆盖率达到36.2%。中心城区和平南城区生活垃圾污水处理厂建成投入使用，桂平城区污水处理工程基本建成。大力开展城市环境综合整治，实施亮化、美化工程，市容市貌有了明显改观。

（七）重视解决群众切身利益问题，改善民生工作取得新成效。大幅度提高民生投入，全市用于解决民生问题的财政支出达38.4亿元，占财政总支出的61.3%，增长25.9%，其中支农惠农投入8.2亿元，增长83%；社保与就业投入4.38亿元，增长22.1%；教育投入19.82亿元，增长21.7%；科技投入0.13亿元，增长14.8%；文化体育投入0.4亿元，增长35.1%；环境保护投入0.91亿元，增长26.2%；医疗卫生投入5.66亿元，增长46.4%。突出抓好为民办实事工程，全面按标准要求完成了自治区政府和市政府提出的为民办实事项目建设任务。积极拓宽就业渠道，新增城镇就业人数1.73万人，下岗失业人员实现再就业4562人，城镇登记失业率控制在3.6%以内。大力推进养老、失业、医疗、工伤和生育保险工作，各类保险人数均超额完成年度任务。提高城乡低保标准和救助水平，新建、改扩建23所乡镇敬老院。启动城镇居民基本医疗保险，参保人数31.2万人。提高新型农村合作医疗标准，全市参合农民393万人，参合率达91.1%。加大住房保障力度，投资1.1亿元建成经济适用房8万平方米；加快廉租住房建设，南江点工程竣工并投入使用，西江农场点基本建成主体工程。扎实推进扶贫开发工作，第二批整村推进的29个村通过了自治区验收，减少贫困人口4.1万人。

（八）积极实施科教兴市和可持续发展战略，各项社会事业协调发展。加强创新型城市建设，扎实推进第四轮科技创新行动，与科技厅签订会商制度，构建新的科技支撑平台；引进科技项目5个,获得专利授权108件,贵港动力、源安堂、安泰化工等3家企业被认定为广西制造业信息化示范企业。投入1.77亿元改造和新建中小学校舍16.72万平方米。中小学校教育资源进一步整合优化，职业教育攻坚稳步推进，其他各级各类教育都取得新发展。卫生工作得到加强，手足口病、甲型H1N1流感等重大疫病得到有效防控，完成17个基层医疗卫生机构的基础设施改造。加快公共文化基础设施建设，建成9个乡镇综合文化站。群众性体育活动蓬勃开展,竞技体育取得新成绩。稳步推进人口和计划生育工作,年度人口出生率为13.98‰，低于自治区下达控制指标2个千分点。国防动员和民兵预备役工作进一步加强，双拥共建活动成效显著。民族、宗教、地方志、对台、统计、物价、广电、公积金、人防、气象、地震、档案、老龄、残疾人以及其他社会事业都取得了新的成绩。

（九）加强社会主义精神文明和民主法制建设，社会和谐稳定。大力开展精神文明创建活动，城乡文明程度不断提高，我市荣获“自治区文明城市”、“中国最佳投资环境城市”称号。文化市场管理得到加强，新闻出版业健康发展，“扫黄打非”行动取得新成效。普法工作有序推进。自觉接受人大法律监督和政协民主监督，办理人大代表建议61件、政协委员提案236件。密切联系工会、共青团、妇联等人民团体，广泛听取社会各界意见和建议。认真贯彻落实《政府信息公开条例》，率先在全区将中央扩大内需重大项目纳入电子监察系统进行监管，并实现了自治区、市、县三级网络对接，行政效能得到进一步提高。认真落实党风廉政建设责任制，强化对行政权力的监督，监察和审计工作得到加强。扎实开展产品质量和食品药品安全专项整治，确保群众食品药品安全。深入开展安全生产隐患排查治理，加强交通、消防等专项整治，安全生产形势总体平稳。进一步健全应急机制，应急水平有了新的提高。认真开展“大接访”活动，实施“维稳基石工程”，精心组织“严打”斗争，深入排查化解矛盾纠纷，社会治安秩序良好，在全国“两会”、新中国成立60周年庆典、第六届中国—东盟博览会等重大节庆活动期间，没有出现上京赴邕的非正常上访现象。

各位代表，面对复杂多变和严峻的经济形势，面对经济社会发展中的诸多矛盾和问题，我们深感成绩来之不易。这是自治区党委、政府和市委正确领导的结果，是全市干部群众团结一心、奋力拼搏的结果。在此，我代表市人民政府，向辛勤工作在各条战线的广大干部群众致以崇高的敬意！向大力支持政府工作的人大代表、政协委员、各民主党派、工商联、各人民团体、离退休老同志和社会各界人士，向积极参与贵港建设的驻贵中区直单位干部职工、驻贵部队和武警官兵，向关心贵港发展的海内外朋友，表示衷心的感谢！

在看到成绩的同时，我们也清醒地认识到，我市经济社会发展还存在不少困难和问题。主要表现在：经济运行中一些突出问题和深层次矛盾依然存在，重大项目支撑拉动作用不够强，企业抵御市场风险能力较弱；产业结构有待优化，科技创新水平有待提高，现代服务业、高新技术产业发展不快；农业基础设施建设滞后，农业产业化水平较低，农民增收的制约因素较多；资源环境约束加剧，节能减排压力较大；关系群众切身利益的就业、住房、医疗、教育等民生问题有待更好解决；改革发展中出现的矛盾纠纷较多，安全生产监管还存在薄弱环节，维护社会稳定的任务仍然较重；政府自身建设需要进一步加强，社会管理和公共服务能力有待进一步提高。对这些问题，我们要主动作为，攻坚克难，全力破解，推动经济社会持续快速健康发展。

2010年工作安排

2010年是实施“十一五”规划和科学发展三年计划的最后一年。做好今年经济社会发展工作，对夺取应对国际金融危机冲击全面胜利、保持经济平稳较快发展、为“十二五”规划启动实施奠定良好基础具有十分重要的意义。纵观宏观形势，审视我市发展，我们既面临着巨大的压力，也迎来更大的机遇。一方面，世界经济复苏基础并不稳固，国际金融危机影响仍然存在，全球性挑战压力增大。虽然我国经济已有所回升，但回升的基础还不牢固，不确定因素还很多，积极变化和不利影响同时显现，短期问题和长期问题相互交织，国内因素和国际因素相互影响，保持经济平稳较快发展、推动经济发展方式转变和经济结构调整难度加大。我们必须做好应对更加困难局面、迎接更加激烈竞争的思想准备。另一方面，国家保持宏观经济政策的连续性和稳定性，继续实施积极的财政政策和适度宽松的货币政策，实施十大产业振兴一揽子计划，出台《关于进一步促进广西经济社会发展的若干意见》，把建设西江经济带纳入国家发展规划，同时自治区作出加快西江“黄金水道”建设和打造桂东四市承接产业转移示范基地的决策，出台做大做强做优工业以及一系列产业调整振兴计划，这些都为我们带来了扩大投资、调整结构、增强后劲、改善民生的政策叠加机遇。从贵港自身来说，经过多年坚持不懈抓项目，全市一批支撑力和带动力较强的重大项目启动实施，拉动经济增长的效应将逐步显现，完全具备了更好更快发展的坚实基础和条件。综观全局，只要我们用好机遇、坚定信心、沉着应对、乘势而上，在更高层次上吸纳更多优质要素，就一定能够把政策优势转变为经济优势、资源优势转变为产业优势、后发优势转变为发展优势，就一定能够战胜困难，实现经济社会平稳健康发展。

2010年政府工作的总体要求是：全面贯彻党的十七大和十七届三中、四中全会精神，以邓小平理论和“三个代表”重要思想为指导，以科学发展观统领全局，按照继续保增长、保民生、保稳定、保持和扩大经济社会发展良好势头以及农民人均纯收入“三年赶超”目标的要求，坚持“六靠”工作思路不动摇，紧紧抢抓政策机遇，采取更加积极有效的措施，坚持扩大经济总量与优化经济结构、转变发展方式相结合，产业培育与园区建设相依托，增加投资与扩大内需相促进，城市发展与农村发展相统筹，经济建设与社会建设相协调，进一步加快发展步伐，着力保障和改善民生，实现经济社会更好更快发展。

2010年国民经济和社会发展的预期目标为：地区生产总值增长12%，全社会固定资产投资增长30%，财政收入增长13%，社会消费品零售总额增长18%，城镇居民人均可支配收入增长10%，农民人均纯收入增长12%，居民消费价格指数涨幅控制在5%左右，化学需氧量和二氧化硫排放总量分别削减9.7%、10.75%，城镇登记失业率控制在4%以内，人口自然增长率控制在9‰以内。

围绕上述目标任务，我们将重点抓好以下工作：

（一）优化产业结构，加快提升产业层次

充分利用建设西江经济带、打造西江黄金水道和构建产业转移示范基地等政策叠加机遇，把促进转变发展方式、增强经济竞争力作为应对挑战、保持经济增长的主攻方向，加快调整优化经济结构，构筑现代产业体系，提高经济增长质量和效益。

培育工业新优势。认真对接中央和自治区实施的产业调整振兴计划，坚持走新型工业化道路，提高自主创新能力，推动产业集聚，增强发展后劲。重点抓好建材、冶金、化工、机械、糖纸等传统产业的优化升级，整合提升羽绒、服装、食品、农产品加工等特色产业，大力发展电力能源产业，积极构建具有较强竞争优势的特色产业链。坚持企业改造升级和培植新型产业相促进，引进新技术、新设备、新工艺，支持清隆机械、鑫发冶炼、宏源矿业、鑫益饲料等规模企业扩能改造和产品开发。力争年内实现建材产业产值超70亿元，冶金产业产值超60亿元，电力能源产值超50亿元，化工、机械、农产品加工三大产业产值分别超30亿元；产值超亿元的企业达60家，其中超10亿元企业10家。落实项目推进负责制，全力推进188个规模以上产业项目，力争完成投资128.2亿元，重点抓好台泥粉磨站、金茂化工、华润 (桂平）一期、益海嘉里粮油加工、利维造船等项目建设，继续推进桂平陶瓷城、粤港皮革城项目，规划建设中港工业城。加大政策扶持，及时掌握煤、电、油等原材料和产品市场信息，综合运用调控措施，提高工业抗风险能力，力争规模工业增加值增长25%以上。

大力发展现代服务业。把发展第三产业作为调整经济结构、扩大内需的重要任务，促进服务业发展提速、比重提高、层次提升。积极构筑现代物流体系，规划建设北煤南调物流基地、钢铁中转基地项目，抓好金泰物流、丰宝物流等一批服务业重点项目，跟踪落实松宇国际机电机械综合市场、澳门商业街、台湾大润发超市等项目。扶持发展骨干物流企业，壮大交通运输业。继续推进“万村千乡”市场工程、“双百”市场工程、“新网”工程和“农超对接”工程，大力发展连锁经营、物流配送，拓展农村、社区连锁销售终端网络。落实“家电下乡”、“汽车下乡”等鼓励消费的政策措施，提高居民的消费预期和信心，扩大即期消费规模。支持发展金融、保险、证券、信息等生产性服务业，完善社区服务设施、服务网络，拓展社区服务领域。编制全市旅游发展总体规划，进一步整合旅游资源，改善旅游设施条件，加强旅游宣传和推介，力争全年接待游客、旅游总收入分别增长15%和30%，逐步把旅游业培育成为重要的战略性支柱产业。

加大节能减排力度。大力发展低碳经济，鼓励和支持企业积极推进新上节能项目的开工建设和投产达效，加强资源综合利用。加快江南、桂平城区生活垃圾污水处理工程建设，规划建设覃塘城区生活垃圾污水处理工程。继续通过限期治理、挂牌督办等措施推进重点污染治理工程建设，促进生态和谐。

扶持发展中小企业和非公经济。坚持把发展民营中小企业作为调整经济结构、扩大城乡就业的重要措施。认真落实国家支持中小企业发展的各项政策，大力扶持中小企业开展技术改造和科技创新，提升产品科技含量和市场竞争力。积极为中小企业发展提供融资服务，协调金融机构扩大对各类中小企业的贷款规模。鼓励和扶持非公资本进入国家法律、法规没有明确禁入的行业和领域，激活民间资本。

（二）突出农民增收，加快发展现代农业

坚持以实现农民人均纯收入“三年赶超”目标为抓手，稳步推进农村改革，加快发展农村公共事业，促进农业增效、农民增收、农村发展。

大力发展现代农业。在稳定粮食播种面积401万亩、产量145万吨的基础上，扶持壮大优势种植业，重点抓好木薯、桑蚕、中草药、蔬菜等特色农产品的基地化、集约化、标准化、信息化和品牌化建设，大力发展城郊型农业、休闲观光农业，力争特色农作物种植面积达580万亩，实现产值60亿元，种植业农民人均现金收入超过1800元。把建设和发展标准化规模养殖场（小区）作为转变畜牧水产业生产方式的重点，新建标准化养殖示范场100个以上，新发展生猪规模养殖场100个以上。抓好重大动物疫病防控，确保畜牧水产业健康发展。

多渠道增加农民收入。加大扶持力度，支持农业龙头企业做大做强，力争新增农业龙头企业6家以上。引导和支持龙头企业采取订单农业、入股分红等方式，密切并规范企业同农民、农业生产的联系，增加农民收入。积极发展农民专业合作组织，年内力争新增农民专业合作社130家，并充分发挥其辐射带动作用。积极引导和鼓励农民就地转移就业、外出务工、回乡创业。争取年内培训农民40万人次，引导和组织外出务工94万人，实现务工收入85亿元。落实农业各项财政补贴和涉农税费减免优惠政策，多方促进农民变现增收。

改善农民生产生活条件。抓住国家新一轮鼓励和促进农村经济发展的机遇，积极争取项目和资金，加强农业和农村基础设施建设，有效改善农业发展环境和村容村貌。抓好达开水库、岭蒙水库等6座大中型水库的除险加固，加快实施达开、武思江、六陈等三大水库灌区的续建配套与节水改造工程，新建一批节水灌溉、渠系改造等节水项目，完成渠道硬化43.6公里，恢复农田有效灌溉面积9600亩，改善灌溉面积18.6万亩。投资2.25亿元修建81个乡村公路项目，实现100%行政村通等级以上公路，其中通村水泥路比例达60%以上。投入1.8亿元加快新一轮农村电网改造，提高农村供电可靠率。切实抓好扶贫开发，年内实现5万人以上脱贫目标。落实农机具购置补贴，加大农机推广力度，不断提高农业机械化水平。

（三）精心打造西江经济带，构建经济发展新格局

抢抓西江经济带建设纳入国家区域发展战略的机遇，坚持“江海联动、港城互动”发展思路，认真抓好西江经济带的规划，加快平台构建，加速项目推进，全面提升沿江开发的层次和水平。

加快贵港港开发建设。围绕西江黄金水道的建设规划和贵港港总体规划，多渠道筹措资金，启动建设猫儿山作业区三期工程、东山多用途泊位工程等项目，加快续建桂平航运枢纽二线船闸、罗泊湾作业区20万标箱及件杂货配套堆场和库场项目、猫儿山作业区二期工程、郁水作业区仓储码头工程、武林港二期工程等项目，推进贵港至梧州一级航道、来宾至桂平二级航道、贵港至南宁二级航道、贵港航运枢纽二线船闸、桂平东塔作业区和龙门陶瓷园区码头等项目的前期工作，进一步提升贵港港江海联运、铁水联运、水陆联运能力，发挥港口的门户和引领功能。扶持发展航运、港口物流、集装箱联运、船货代理等中介服务机构，适时增加开通贵港直航目的港班轮航线，吸引本地和更多的外地货物从贵港港进出，增加港口货物吞吐量，力争全年港口货物吞吐量实现3700万吨以上，其中集装箱吞吐量达到7万标准箱以上。加快贵港港口岸服务设施建设，深化港口管理体制改革，提高口岸信息化管理水平，提供更加便捷的查验通关服务。加大标准化船型推广力度，促进船舶运力结构调整和优化。加强水上交通安全保障体系建设，提高航道运输安全管理水平。

增强园区承载能力。按照“竞争力强、集约度高、配套性好”的要求，加快沿江产业园开发建设，完善园区基础设施，提升园区功能配套能力。突出抓好市江南工业园区基础设施建设，优化服务环境，努力成为沿江经济发展的主战场。力争贵港（台湾）产业园、桂平龙门工业集中区、平南临江工业园成为自治区A类产业园区。在推进园区建设过程中，各县市区都要精心策划，发挥优势，突出重点，抓出成效。港北区重点抓好商贸、物流、能源产业，加快西江产业园区建设；港南区重点抓好羽绒、化工、木业集中区建设和劳动密集型产业；覃塘区重点抓好台湾（贵港）产业园区建设和建材、物流、农产品加工业发展；桂平市重点抓好长安工业园区建设和冶矿、机械、食品、服装针织、旅游、制药、造船和陶瓷业发展；平南县重点抓好临江产业园区建设和建材、化工产业发展。总之，要进一步明晰县市区及所属园区的产业发展定位，促进产业集群、企业集聚，形成错位发展、优势互补、各具特色的产业发展新格局。

发展临江大产业。把发展大型临港临江产业作为西江开发的重中之重，努力培育新的增长点。重点推进建材、电力、化工、冶金、机械等重大产业以及产业链项目建设，加快培育造船、物流等新兴产业和临港物流园区，积极推进大藤峡水利枢纽工程、平南白沙核电项目前期工作，全力打造先进制造业基地和区域性港口综合物流枢纽。有效对接国家战略和自治区出台的重大举措，从战略层面策划、编制一批事关长远的重点事项，促进一批多年来努力争取的重大产业项目和发展事项列入国家和自治区计划，实现早日开工。

（四）统筹城乡协调发展，着力打造宜居宜业环境

着眼宜居宜业、城乡互动，加快城乡一体化进程，促进城市空间布局优化、承载能力提升、服务功能完善，增强城市承载力、带动力和辐射力，力争城镇化率达到39.2%以上。

完善城乡规划体系。搞好城北新区二期、港南片区控制性规划和南湖公园专项规划，力争中心城区建成区面积达51平方公里以上，进一步拉开城市框架，提升城市品位。指导各县市区完成总体规划修编和控详规划编制，搞好重点建制镇规划，形成与中心城市功能互补、规模适度的城镇体系。建立完善规划决策机制，开展规划执行监察，坚决制止和查处违章违法建设行为。

增强中心城区承载力和吸纳力。开工建设市体育中心、文化艺术中心、档案馆和民族文化公园、南湖公园。加快推进市图书馆、博物馆、东湖整治工程、郁江南北堤路园建设。继续抓好建设西路、桂林路、仙衣路北延段、中山路北延段等城市道路建设，新开工郁林路、金田路、民主路、浔州路、龚州路、贵州路、城北大道、解放路中段、布山大道、同济大道等城区道路，进一步完善城区路网，缓解城区交通压力。规划建设市区农贸市场、农副食品批发市场等公共服务设施，进一步增强城市综合服务功能。强化政策引导，扩大居民住房消费，促进房地产业稳定健康发展。

提升城市管理水平。进一步建立健全城市市政公用事业各行业管养分离的工作机制和“两级政府、三级管理、四级网络”的城市管理工作机制，继续加强精细化管理。深入拓展“城乡清洁工程”和市容环境综合整治“南珠杯”竞赛活动，加大环境卫生、市容市貌、交通秩序、市场秩序整治力度，努力营造更加适宜发展创业、更加适宜人居、文明和谐的城市环境。

着力完善城乡基础设施。努力增加对城乡饮水、电力、道路、通信、垃圾处理设施等方面的建设投入，逐步实现城乡共建、城乡联网、城乡共用。全力配合推进南广高速铁路和梧州至贵港、桂平（石龙）至来宾高速公路（贵港段）建设，力争开工建设贵港至合浦高速公路（贵港段）、荔浦至玉林高速公路（贵港段）、贵港东环路郁江特大桥和平南武林港进港公路，抓好柳州至平南高速公路（贵港段）、贵岑二级公路港南至四灵段改建工程的前期工作。以提高水利防灾减灾能力为目标，切实加强农村安全饮水、水库除险加固等水利基础设施建设。投入10亿元加快推进逢宜2#主变扩建、国茂等重大输变电工程，满足经济发展和市民的用电需求。坚持把县城、小城镇、居民点建设与市场建设、产业集聚发展结合起来，增强城镇综合经济实力和辐射力，引导人口向城镇转移、产业向园区集中，以人口和产业集聚带动县城规模扩大和中心城镇发展。

实施城乡风貌改造工程。按照“政府引导、群众参与、规划先行、示范带动”的原则，全面启动城乡风貌改造工程，重点抓好试点村屯和沿街、沿路建筑物的立面改造。大力开展造林绿化，突出抓好西江沿岸、国道路旁、村屯屋边的造林，改善城乡人居环境。

（五）加强财政金融工作，支持经济社会发展

坚持生财、聚财、理财并重，进一步完善公共财政体制，优化金融环境，为经济社会发展提供强有力的支撑。

加快财源建设。进一步完善财源建设目标考核管理制度，做好基础财源企业的跟踪服务工作。用足用活财政政策支持优势产业发展，巩固提升电力、建材、冶金、糖纸等骨干税源，扩大中小税源，培育新兴税源，努力壮大税源基础。

强化财税征管。实施税源动态管理，加强对重大项目、重点企业、重点税种的税收征管，确保重点税源和主体税种收入稳定增长；坚持严征细管，加大对零散税源和易流失税收的征管力度，做到依法征收、应收尽收。抓好非税收入的征收管理，严格税费减免政策。

优化支出结构。强化预算约束，严格控制一般性支出，在确保法定支出的基础上，加大对“三农”、教育、医疗卫生、社会保障等方面的投入，集中财力为民办实事、解难事。进一步完善和加大转移支付力度，努力缓解县乡财政困难。强化重点项目、重点资金审计监督，提高财政资金使用绩效。

改善金融服务环境。进一步完善政银企沟通协调机制，搭建合作平台，找准产业发展与信贷支持的结合点，为地方经济发展提供强有力的金融支撑。推进信用体系建设，强化企业的信用评价和监督管理。支持金融机构拓展经营领域，扩大信贷规模，加大对重点工业项目、中小企业、节能减排、新农村建设、产业升级等重点领域和薄弱环节的信贷支持，最大限度地增强金融服务发展的能力。扩大保险覆盖面，提高保险服务水平和防范风险能力。

（六）坚持深化改革，全面提升对外开放水平

稳步推进重点领域和关键环节的改革，全面扩大对外开放和区域合作，增强经济社会发展的动力和活力。

全面推进改革创新。在保持农村现有土地承包关系稳定不变的基础上，建立健全县乡土地流转管理和服务机构，鼓励农民按照“依法、自愿、有偿”的原则，以转包、出租、互换、转让、股份合作等多种形式流转土地承包经营权，加快土地流转改革，推进土地适度规模经营。因地制宜探索户籍制度、社会保障、就业服务等方面改革，加快推进城镇化。抓好城乡建设用地存量挖潜改造，实行最严格的土地管理制度和耕地保护制度。大力推进集体林权制度改革，力争年内基本完成主体改革任务。大力发展农业保险，支持供销合作社、邮政服务点、农民专业合作社、专业服务公司、专业技术协会、农民经纪人、龙头企业等为“三农”提供多种形式的生产经营服务。深化国企改革，巩固国企改革成果。继续深化粮食、供销等流通企业改革，不断增强发展活力。进一步强化财政职能，完善事权与财力相匹配的财政体制。深化国库集中收付、政府采购和投资评审管理制度改革，加强财政资金筹集、分配、管理的全过程监督。深化农村信用社改革，解决农村融资难问题。全面完成市、县两级政府机构改革，加快事业单位分类改革。

不断提高开放型经济水平。坚持专业化、市场化、园区化、产业化招商引资的路子，突出引进战略投资者和大项目。优化招商引资项目库，增强项目投资吸引力。加大产业、基础设施和服务业项目的招商力度，不断拓宽引资领域。充分发挥人文、资源、区位优势，密切同珠三角、长三角、港澳台等重点地区的联系，加快承接沿海发达地区产业转移。强化软环境建设，努力营造“亲商、富商、安商”的发展氛围，确保实际利用市外资金增长20%、直接利用外资增长15%以上。认真落实国家和自治区扶持外贸出口的政策，进一步壮大出口主体，鼓励各类企业开展对外贸易，力争实现外贸进出口总额增长10%以上。

大力推进全民创新创业。支持返乡农民工、大中专毕业生和其他新增就业群体自主创业，积极引进市外投资主体来贵港创业，发挥大中型企业在创业中的引领带动作用。聚集创新资源，鼓励支持社会各界开展科技创新，引导企业研究引进应用新技术、开发新产品，提高核心竞争力。

（七）加强社会管理，推进以改善民生为主的社会建设

坚持把改善民生、发展社会事业作为发展经济的最终目的和扩大内需、调整经济结构的重点，认真落实各项民生政策，加快发展各项社会事业，提高公共服务水平，让改革发展的成果普惠于民。

加强就业和社会保障，提高城乡人民生活水平。实施更加积极的就业政策，统筹做好返乡农民工、大中专毕业生、被征地农民、城镇新增劳动力、农村富余劳动力和城乡就业困难群体的就业指导和服务工作，鼓励创业带动就业,力争新增城镇就业人数1.6万人,新增农村劳动力转移7万人。进一步扩大养老、失业、工伤等社会保险覆盖面，健全城镇职工基本医保、城镇居民基本医保和城乡医疗救助制度，提高新型农村合作医疗参合率。继续抓好城乡低保和农村五保提标扩面工作。逐步解决政策性破产国有企业以及关闭破产集体企业退休人员和困难企业职工等城镇未参保人员的医疗和养老保障问题。探索建立农民工养老保险、被征地农民养老保险和农村养老保险制度。大力发展慈善事业，提高社会救助水平。通过发展经济，逐步提高居民收入在国民收入分配中的比重和劳动报酬在初次分配中的比重，提高企业退休人员养老金标准和部分优抚对象生活补助标准。

加快发展社会事业。认真组织实施“中小学校舍安全工程”，加大改造和扩建城区学校力度，合理调整中小学校布局，优化整合教育资源，推动义务教育均衡发展。加强职业学院建设，大力发展中等职业技术教育，逐步实行中等职业教育免费，提高教育服务经济社会发展的能力。重视学前教育，关心特殊教育，发展远程教育和继续教育，引导和扶持民办教育，关心外出和外来务工人员子女、困难家庭子女的学习和生活。实施“西江创业创新领军人才行动计划”，多渠道引进高层次和紧缺急需专业人才，发挥各类人才的作用。推进医药卫生体制改革，完善卫生监督和农村、社区卫生服务体系建设，加强重大疾病防控工作，发展中医药和妇幼保健事业。深入实施“文化信息共享”、“新农村文化致富”和“文化下乡”工程，丰富农村文化生活。发展文化产业，加强网络文化建设和管理。广泛开展全民健身活动，提高竞技体育水平。健全人口和计划生育工作长效机制，加强流动人口管理服务，稳定低生育水平。加快发展社会科学，繁荣新闻出版、广播电视、文学艺术。加强民兵预备役建设，做好国防动员和双拥共建工作，巩固军政军民团结。依法保障妇女和未成年人权益。统筹发展其他各项社会事业。

促进社会和谐稳定。深入推进“五五”普法教育，加强法律服务和法律援助工作。抓好以社会主义核心价值体系为根本的“和谐建设在基层活动”，提升城乡文明程度。继续实施“维稳基石工程”，落实社会治安综合治理领导责任制，健全社会治安防控体系，依法严厉打击和预防各种刑事犯罪。加强信访工作，完善化解矛盾纠纷长效工作机制，预防和有效处置群体性事件。落实安全生产责任制，强化重点行业和重点领域的安全监管，防范重特大事故发生。健全应急管理机制，提高处置突发事件的能力，确保社会和谐稳定。

抓好为民办实事和惠民工程。今年要认真办好以下实事：①实施城乡风貌改造工程，完成12个自治区级城乡风貌改造工程示范点建设任务；实施市中心城区金港大道东西两个主要出入口和中山路中段、建设路北门市场路段的改造；桂平、平南分别完成2个主要道路出入口、各县市区分别完成二级公路沿线2个村屯的改造任务。②在中心城区建成并投入使用荷城初中，新建、改建或扩建三所中学和三所小学，筹建一所特殊教育学校，进一步缓解城区居民子女入学难问题。加大改造中小学校危房工作力度，进一步改善中小学校舍安全状况。③在中心城区新区规划筹建一所综合性医院，缓解城市居民就医难问题。④规划筹建市老年活动中心、青少年活动中心和妇女儿童活动中心，为广大老年人、青少年和妇女儿童提供良好的活动场所。⑤在中心城区建设街头绿地3个、公厕10座、垃圾转运站3座，在主要街道和公园景区安装休闲椅1000张、添置果皮箱2000个；完成旧城区150条小街小巷1700盏路灯改造安装、硬化路面100条2.5万平方米、改造排水沟渠30条4.5公里，基本搞好旧城区现有小街小巷改造，进一步优化城市人居环境。⑥新建96个五保村，改善农村五保户集中供养条件。⑦年内新建廉租住房2万平方米400套，规划建设经济适用房1558套，逐步解决城镇低收入家庭住房难问题；加大农村危房改造支持力度，积极筹措资金帮扶贫困农民改造危房。⑧筹资3900万元修建建制村通公路项目11个83公里，其中通村水泥路项目9个75公里，实现全市行政村全部通四级以上公路；投资7500万元，建设水库移民进村（屯）道路工程110个，改建移民新村75个，让10万库区移民和10万非库区群众受益。⑨筹资1500万元，补助新建5000座沼气池；筹资1.25亿元实施人饮安全工程100个，解决农村25万人的饮水不安全问题。⑩搞好18个乡镇的综合文化站建设，新建农家书屋245家，丰富农村群众文化生活。

（八）加强自身建设，提高政府管理和服务水平

新的形势和任务，对政府工作提出了更高的要求。实现新一年的经济社会发展目标，政府必须切实加强自身建设，全面提高服务水平和执行力。

转变政府职能，提高办事效率。完成新一轮政府机构改革，优化组织结构，理顺部门关系，强化公共服务职能，完善经济调节机制。继续精简行政审批事项，下放经济社会管理权限，推行项目联审联办，简化办事环节，做到特事特办、急事快办。

坚持依法行政，规范政府行为。自觉接受人大及其常委会的法律监督，主动接受政协的民主监督，认真听取社会各界意见，完善重大事项集体决策、专家咨询、社会公示听证等制度，促进依法行政、科学施政。加强法治理念教育，提高政府工作人员依法行政、依法办事的能力。严格实行首问负责制、限时办结制和责任追究制，做到有错必纠、有责必问。

切实转变作风，狠抓工作落实。大力精简会议文件，大兴调查研究之风，把更多的时间和精力用于深入基层、解决实际问题上。强化政府工作目标责任管理，健全重点工作推进责任机制，对事关全局的重大决策，定时间、定标准、定要求，确保落实到位。凡是市委、市政府的重大决策和工作部署，政府各级各部门都要雷厉风行、不折不扣地贯彻落实。进一步加强督查工作，及时掌握进展情况，高效推进各项工作。强化考核奖惩，加快建立和完善科学合理、行之有效的政府绩效考核评估体系。

坚持从严治政，树立清廉形象。认真落实廉政建设责任制，加大对权力部门、重点行业监督力度，加强对重点专项资金、重大投资项目和工程招投标的审计监察，严肃纠正损害群众利益和破坏投资环境的行为，严厉查处违法违纪案件，树立良好的政府形象。

各位代表，加快发展是形势的需要、时代的强音，富民强市是人民的愿望、政府的职责。新的一年，困难和挑战考验着我们，责任和使命激励着我们。让我们更加紧密团结在以胡锦涛为总书记的党中央周围，深入贯彻落实党的十七大和十七届三中、四中全会精神，在市委的领导下，坚持发展为上、民生为本、务实为先、律己为诫，不辜负全市人民的重托，共同谱写贵港经济社会科学发展、和谐发展、跨越发展的新篇章！
